# Genome-wide association identifies methane production level relation to genetic control of digestive tract development in dairy cows

**DOI:** 10.1038/s41598-018-33327-9

**Published:** 2018-10-11

**Authors:** M. Pszczola, T. Strabel, S. Mucha, E. Sell-Kubiak

**Affiliations:** Department of Genetics and Animal Breeding, Poznan University of Life Sciences, Wolynska 33, Poznan, Poland

## Abstract

The global temperatures are increasing. This increase is partly due to methane (CH_4_) production from ruminants, including dairy cattle. Recent studies on dairy cattle have revealed the existence of a heritable variation in CH_4_ production that enables mitigation strategies based on selective breeding. We have exploited the available heritable variation to study the genetic architecture of CH_4_ production and detected genomic regions affecting CH_4_ production. Although the detected regions explained only a small proportion of the heritable variance, we showed that potential QTL regions affecting CH_4_ production were located within QTLs related to feed efficiency, milk-related traits, body size and health status. Five candidate genes were found: *CYP51A1* on BTA 4, *PPP1R16B* on BTA 13, and *NTHL1*, *TSC2*, and *PKD1* on BTA 25. These candidate genes were involved in a number of metabolic processes that are possibly related to CH_4_ production. One of the most promising candidate genes (*PKD1*) was related to the development of the digestive tract. The results indicate that CH_4_ production is a highly polygenic trait.

## Introduction

The increase in the global temperature has a serious impact on the environment and humans. Some of these consequences may exceed adaptive capacities of some species, lead to water supplies shortage, melt the glaciers and increase sea level as well as trigger extreme climatic events^1^. The estimated global temperature increase in 2010 due to greenhouse gas (GHG) and aerosol emissions was 0.81 °C in relation to the pre-industrial era. The 0.11 °C of this increase was contributed by methane (CH_4_) emissions from direct livestock emissions^2^. Most of livestock CH_4_ emissions are caused by ruminants^3,4^. The CH_4_ emissions from ruminants are mostly due to enteric fermentation.

In ruminants the enteric fermentation is a consequence of a normal digestive process. One of their stomachs, the rumen, is inhabited by rumen microorganisms, enabling digestion of feed that contains high amounts of fiber. One of the by-products of this digestive process converting the feed provided to the ruminants by the microorganisms is CH_4_.

The CH_4_ consists of carbon. When carbon is lost from the body it may no longer be used by the animal as a source of energy. Therefore, apart from its environmental impact, CH_4_ emission in ruminants has also a potential negative impact on the profitability of animal production^5,6^. Due to those potential consequences of CH_4_ emissions from ruminants, mitigation strategies are under investigations. Optional strategies range from adjusting the management to nutritional treatments^7^. These strategies may have a high impact on CH_4_ production. An additional strategy of mitigating CH_4_ production might involve selective breeding for lower emitters. Such a strategy could be possible in case of the existence of genetic variation in CH_4_ production and a favorable genetic association of CH_4_ and traits in the current breeding goals.

Recent genetic studies on dairy cattle revealed that while most of the variation in CH_4_ production is due to non-genetic factors (i.e. feed, management and other environmental factors), the genetic component (i.e. genetic variance) in CH_4_ production also exists^8–13^. However, information on the extent of genetic control over CH_4_ production and the genetic architecture of the trait is generally scant. For example, Manzanilla-Pech *et al*.^14^ performed GWAS on different methane phenotypes in beef cattle and validated the results on dairy cattle, whereas Van Engelen^15^ performed GWAS on Holstein cows using phenotypes predicted from milk and breath analyses_._

Only lately the technology for measuring CH_4_ production both on a large scale and on individual animals has become available. Among others, high throughput measuring techniques are based on breath analyses, since approx. 90% of enteric CH_4_ is released during eructation events and by breathing^5^. Nonetheless, collection of such phenotypic records is still challenging and data sets are limited. This technique is promising as it is non-invasive, based on infra-red analyses of breath samples and measurements can easily be taken during milking or feeding^13,16–19^. The most common application of these techniques is in combination with automatic milking systems (AMS) enabling a relatively long measurement period (duration of the milking); additionally, several observations per cow per day may be collected from a large number of animals. This type of measurement set up enables collection of large volumes of data, which is a prerequisite for genetic analyses.

To our knowledge, to date no reports are available on genome-wide association analyses based on direct measurements of daily CH_4_ production in dairy cattle. Therefore, the objective of this study was to undertake a genome-wide association study using CH_4_ phenotypes measured by breath analyzers to unravel the genomic regions controlling CH_4_ production from dairy cattle.

## Results

### Detected SNPs

**Figure 1:**
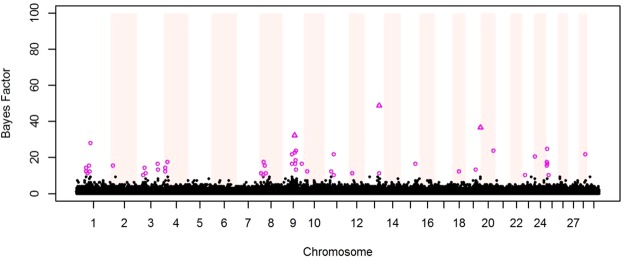
Results of genome-wide association study for raw phenotypic methane production. Pink triangles indicate SNPs with Bayesian Factor (BF) >= 30, pink circles SNPs with 10 =< BF < 30 and black dots non-significant SNPs.

**Figure 2:**
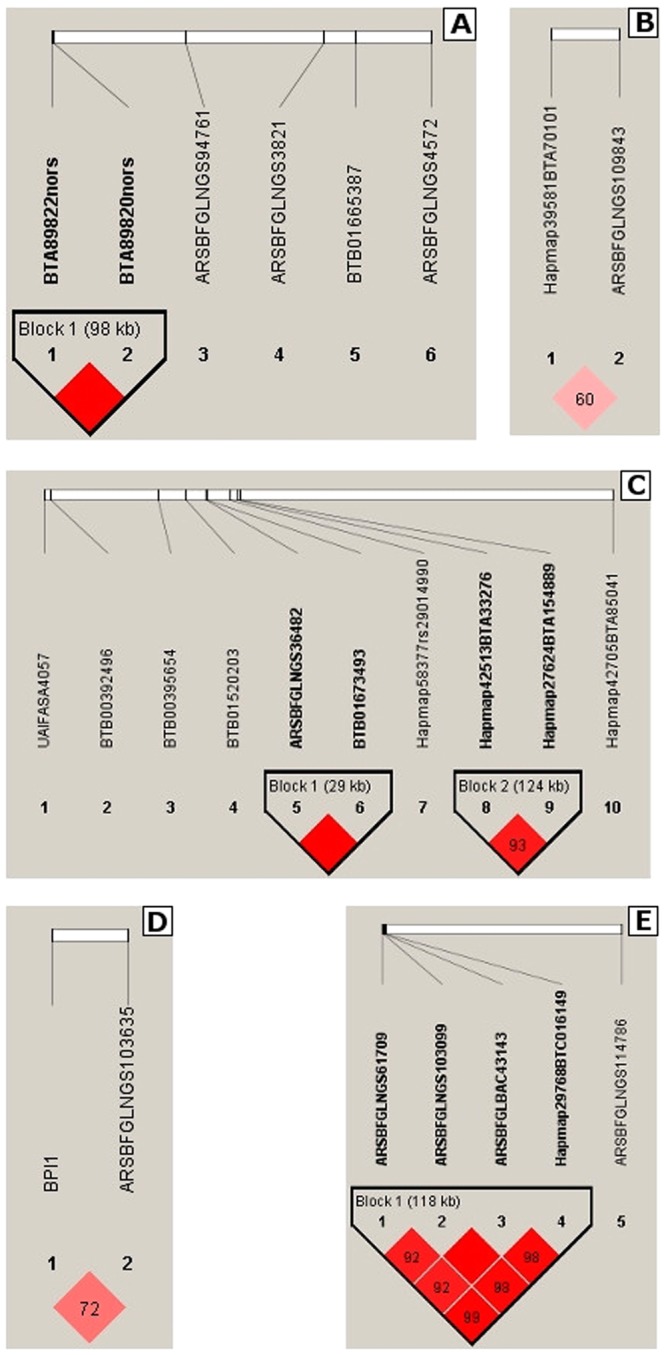
Results of the linkage disequilibrium (LD) analysis for significant SNPs detected on Bos Taurus autosomes (BTA) for raw phenotypic methane production. (**A**) BTA 1, (**B**) BTA 4, (**C**) BTA 9, (**D**) BTA 13, (**E**) BTA 25. Each square contains a value for r^2^ between neighboring SNP.

The genetic variance for daily CH_4_ production was estimated independently for each level of 2^nd^ order Legendre polynomials. As the first parameter explains most of the variation, only SNP detected with it will be presented and discussed in this study. The GWAS performed on daily CH_4_ production indicated 50 SNPs with BF > 10 associated with CH_4_ production in dairy cattle (Fig. 1). Those SNPs were located on 18 different BTA (Tables 1 and 2). From detected SNPs, three had a BF above 30, which is defined as “very strong” association^20^. On BTA 1, 4, 9, 13 and 25 analysis in Haploview^21^ indicated six potential candidate QTL regions (Fig. 2). For those regions and two single SNP associations on BTA 9 and 20, a total of 130 candidate genes (protein-coding and non-coding RNA) were located with BIOMART^22^ (Table 1).

**Table 1:** Candidate QTL regions and single SNPs detected for methane production with Bayesian Factor (BF) above 30, their position in base pairs, minor allele frequency (MAF), number of candidate genes and percentage of total genetic variance explained by them.

BTA	SNP name	Position (bp)	MAF	BF	Candidate QTL	Allele subs. effect^a^	Number of candidate genes	Total genetic var. expl. (%)
1	BTA89822nors	46223040	0.491	14.33	Yes	0.113	12	0.006
1	BTA89820nors	46321775	0.488	12.26		0.193		
4	Hapmap39581BTA70101	9203380	0.497	12.26	Yes	0.226	14	0.003
4	ARSBFGLNGS109843	9615916	0.430	14.33		0.251		
9	BTB00395654	60102040	0.353	32.29	—	0.132	1	0.003
9	ARSBFGLNGS36482	64262480	0.365	16.41	Yes	0.201	9	0.005
9	BTB01673493	64291804	0.365	16.41		0.172		
9	Hapmap42513BTA33276	66997852	0.215	23.76	Yes	0.183	7	0.005
9	Hapmap27624BTA154889	67122449	0.318	13.29		0.158		
13	BPI1	67833218	0.402	11.22	Yes	0.144	21	0.003
13	ARSBFGLNGS103635	67888763	0.467	48.68		0.152		
20	ARSBFGLNGS109784	909076	0.407	36.61	—	0.129	1	0.003
25	ARSBFGLNGS61709	1086505	0.432	17.45	Yes	0.277	65	0.004
25	ARSBFGLNGS103099	1127441	0.395	15.37		0.238		
25	ARSBFGLBAC43143	1184038	0.395	24.82		0.101		
25	Hapmap29768BTC016149	1205232	0.346	16.41		0.321		

^a^Allele substitution effects were estimated as $\alpha =\sqrt{{\sigma }_{a}^{2}{(2pq)}^{-1}}$, where ${\sigma }_{a}^{2}$ is the genetic variance explained by the SNP, and p and q are the frequencies of the two alleles^76^.

**Table 2:** Suggestive SNPs detected for methane production with Bayesian Factor 10 < BF < 30, their position in base pairs, minor allele frequency (MAF) and percentage of total genetic variance explained by them.

BTA	SNP name	Position (bp)	MAF	BF	Allele sub. effect^a^	Total genetic var. expl. (%)
1	ARSBFGLNGS94761	53656600	0.416	11.22	0.088	0.003
1	ARSBFGLNGS3821	61286751	0.337	15.37	0.177	
1	BTB01665387	63061634	0.437	12.26	0.231	
1	ARSBFGLNGS4572	67212088	0.381	28.01	0.179	
2	Hapmap44041BTA23382	10617894	0.266	15.37	0.200	0.001
3	Hapmap33584BTA141202	30922247	0.128	10.19	0.235	0.075
3	Hapmap44183BTA105889	37602383	0.428	14.33	0.172	
3	ARSBFGLNGS38388	43476846	0.421	11.22	0.170	
3	ARSBFGLNGS98870	98587436	0.428	16.41	0.114	
3	Hapmap39765BTA62582	99317016	0.266	13.29	0.225	
4	BTA72259nors	20510260	0.360	17.45	0.172	0.001
8	Hapmap26798BTA82382	11398105	0.191	11.22	0.207	0.012
8	BTB00863195	23634451	0.449	10.19	0.088	
8	ARSBFGLNGS39902	24288969	0.404	17.45	0.114	
8	Hapmap52006BTA77999	29628947	0.449	15.37	0.133	
8	BTB01356348	34847992	0.280	11.22	0.185	
9	UAIFASA4057	50279445	0.245	16.41	0.197	0.005
9	BTB00392496	50899854	0.322	21.65	0.155	
9	BTB01520203	62539556	0.383	22.70	0.166	
9	Hapmap58377rs29014990	66292441	0.353	18.50	0.179	
9	Hapmap42705BTA85041	99135245	0.196	16.41	0.191	
10	BTA59410nors	17730891	0.215	12.26	0.194	0.002
11	ARSBFGLNGS27959	22465305	0.128	12.26	0.161	0.004
11	BTB01397452	33167082	0.428	21.65	0.156	
11	BTB01641011	33771048	0.486	10.19	0.117	
12	BTA31817nors	22219373	0.241	11.22	0.289	0.001
15	ARSBFGLNGS86665	67556240	0.227	16.41	0.196	0.003
18	ARSBFGLNGS14182	33602408	0.323	12.26	0.144	0.001
19	Hapmap48676BTA18047	47374363	0.490	13.29	0.094	0.003
20	ARSBFGLBAC36856	63407185	0.356	23.76	0.129	0.001
23	Hapmap61132rs29019650	11907305	0.402	10.19	0.158	0.001
24	ARSBFGLBAC31288	4273189	0.178	20.60	0.242	0.002
25	ARSBFGLNGS114786	7952738	0.400	10.19	0.141	0.002
28	BTB00987935	35294673	0.400	21.65	0.169	0.005

^a^Allele substitution effects were estimated as $\alpha =\sqrt{{\sigma }_{a}^{2}{(2pq)}^{-1}}$, where ${\sigma }_{a}^{2}$ is the genetic variance explained by the SNP, and p and q are the frequencies of the two alleles^76^.

The three SNP detected for raw phenotypes with BF > 30 and six possible candidate QTL regions explained 0.032% of the total genetic variance (Table 1), whereas the remaining SNPs with 10 < BF < 30 explained 0.122% of this variance (Table 2). Overall this gives a very low result of 0.154% of the total genetic variance explained by detected SNPs.

### Bioinformatics analysis of detected regions

Out of 130 candidate genes for CH_4_ production, 46 remained for a further GO Term analysis as known and non-ambiguous genes. For possible candidate genes, 428 different GO Terms were described: 82 cellular component terms, 251 biological process terms and 95 molecular function terms. Based on the GO Terms, five candidate genes were selected as the most promising: *CYP51A1* on BTA 4, *PPP1R16B* on BTA 13, and *NTHL1*, *TSC2*, and *PKD1* on BTA 25 (Table 3).

**Table 3:** GO Terms for most promising candidate genes detected for methane production in dairy cattle.

Gene	BTA	Position	Type of GO Term	GO Term name
*CYP51A1*	4	9306414–9323252	Biological process	lipid metabolic process
				steroid metabolic process
*PPP1R16B*	13	68258627–68366080	Biological process	establishment of an endothelial barrier
				positive regulation of blood vessel endothelial cell proliferation involved in sprouting angiogenesis
*NTHL1*	25	1590252–1595934	Biological process	metabolic process
*TSC2*	25	1596730–1626967	Cellular component	Lysosome
*PKD1*	25	1627978–1666088	Biological process	blood vessel development
				nitrogen compound metabolic process
				digestive tract development

Based on Cow QTLdb^20^, 52 QTLs involved in production and reproduction traits were selected as potentially playing a role in daily CH_4_ production in cows. Those QTLs were clustered into five groups: feed efficiency, milk related, body size and health status (see Table 4).

**Table 4:** Previously detected QTLs within the identified genomic regions potentially related to methane production.

Group of traits	Trait	BTA
Feed efficiency	Residual feed intake	4
	Feed conversion ratio	4
	Average daily gain	4
Body size	Height (mature)	4
	Chest depth	9
	Body weight (mature)	9
Milk	Milk fat yield	9; 25
	Milk protein yield	1; 13; 20
	Milk yield	13
	Milk energy yield	9
	cis-Vaccenic acid content	20
	Docosatetraenoic acid content	9
	Eicosapentaenoic acid content	9
	Linoleic acid content	1
	Milk alpha-casein percentage	1
	Milk capric acid percentage	13
	Milk caproic acid percentage	13
	Milk caprylic acid percentage	13
	Milk myristoleic acid percentage	13
	Milk palmitoleic acid percentage	1; 13
	Oleic acid content	1
	Polyunsaturated fatty acid content	1
Health status	Somatic cell score	4; 9
	Clinical mastitis	9
	Immunoglobulin G level	4; 20
	Infectious bovine keratoconjunctivitis susceptibility	1; 20
	M. paratuberculosis susceptibility	20

## Discussion

To our knowledge this is the first GWAS on direct measurements of daily CH_4_ production performed in dairy cattle. So far one GWAS on direct measurements of CH_4_ production was performed in beef cattle with validation on dairy cattle^14^ and for CH_4_ intensity^15^. Another GWAS study on dairy cattle^23^ used predicted CH_4_ following the formula proposed by Dijkstra *et al*.^24^. Thus very little is still known on the actual genomic architecture of CH_4_ production in dairy cattle. Our results provide more insight into the genomic architecture of CH_4_ production thanks to the identification of genomic regions involved in the control of this trait and revealed genomic relationships between CH_4_ production and other traits.

Methane emission may be expressed in several ways depending on the aim of a given study^25–28^. First of all, when the total CH_4_ emitted by cows is of interest, the CH_4_ production phenotype expressed in g/d or l/d may be used^16–18,28^. When the goal is to minimize the amount of CH_4_ emitted from the supplied unit of feed (i.e. dry matter intake) in order to maximize feed conversion, the CH_4_ yield^26^ is the trait of interest (CH_4_ produced per kg of dry matter intake). Another way of expressing emission is CH_4_ intensity^26^, where produced CH_4_ is expressed per unit of product (milk or meat). Similarly to the residual feed intake, CH_4_ may be expressed as a difference between predicted and measured CH_4_ emission (i.e. residual CH_4_ emission)^14,26,29^. For our analyses we have decided to use the phenotype applied most widely in the literature and the least influenced by other traits not strictly related to CH_4_ emission itself (e.g. dry matter intake, milk production, live weight). Another reason is related with the fact that when calculating our CH_4_ production phenotype, we account for body weight, physiological status and milk production as described in Pszczola *et al*.^13^ following Madsen *et al*.^28^. Therefore, calculations of CH_4_ yield or CH_4_ intensity may have resulted in some potential overestimation of CH_4_ emissions due to double counting.

### Selected candidate regions

Based on the bioinformatics analysis of detected regions for CH_4_ production in dairy cattle, five most promising candidate genes were selected based on GO Term analysis (Table 3). The first of them, *CYP51A1* (BTA4: 9,306,414-9,323,252) located within the region of a candidate QTL on BTA 4, is a member of the cytochrome P450 family 51 subfamily A. Based on GO Terms this gene is involved in two biological processes that could potentially affect CH_4_ production in dairy cattle. Those GO Terms are the lipid metabolic process and the steroid metabolic process^30,31^, which are confirmed by *CYP51A1* and its family members being involved in the synthesis of cholesterol, steroids and other lipids^32^. Lipids (i.e. fatty acids) were previously reported to be related to CH_4_ production, including several studies that used fatty acids present in milk to predict CH_4_ production^24,33–39^.

The second gene, namely *PPP1R16B* (BTA13: 68,258,627-68,366,080), a protein phosphatase 1 regulatory subunit 16B, is located within the candidate QTL region on BTA 13. For this gene two biological processes were found in GO Terms analysis that could link it to CH_4_ production. One of them, the establishment of the endothelial barrier, e.g. in the intestine, is defined as “… specific and selective control over the passage of water and solutes, thus allowing formation and maintenance of compartments that differ in fluid and solute composition”^40^. The other, the positive regulation of blood vessel endothelial cells^30,40,41^. The biological processes involving *PPP1R16B* suggest that this gene could affect the digestive process by controlling the passage of water within the intestine and providing blood vessels to the endothelial cells of the intestine. Being part of such processes, PPP1R16B could affect efficient use of feed and in this way control the amount of by-products (including CH_4_) produced during the process of digestion.

The three other genes were all located within the largest detected candidate QTL region on BTA 25, comprising of four SNPs. The first of the genes, *NTHL1*, nth like DNA glycosylase 1, is located at 1,590,252-1,595,934 bp. Its GO Term is the metabolic process, which includes protein synthesis and gradation^31,40^. The process involving this gene suggests that *NTHL1* may affect digestive processes and consequently also a number of their by-products, e.g. CH_4_, being released post feeding.

The second of the above-mentioned genes, *TSC2* (BTA25:1,596,730-1,626,967), tuberous sclerosis 2, is the only candidate gene with a GO Term related to a cellular component, in that case lysosome^31,40^. Moreover, *TSC2* has been very well studied in humans, as its mutation causes tuberous sclerosis and its product is believed to be a tumor suppressor^32^. In the case of dairy cattle the location of the *TSC2* gene in lysosome, which contains hydrolytic enzymes and takes part in energy metabolism, suggest that it could be involved in digestion processes and degradation of metabolites, this may affect CH_4_ produced by a cow.

The last of the candidate genes on BTA 25 is *PKD1*, encoding polycystein 1, a transient receptor potentially involved in channel interacting (1,627,978-1,666,088 bp). Three biological processes were assigned to it in GO Term analysis, i.e. blood vessel development^40^, nitrogen compound metabolic process^31,40,42,43^ and the digestive tract development^40^. All three GO Terms indicate that *PKD1* is involved in digestion processes either directly by affecting the development of the digestive tract, or possibly also blood vessels around it as well as metabolic processes of a nitrogen compound. All those functions, in general, indicate that *PKD1* might be involved in emissions of greenhouse gases, not only CH_4_ but also nitrogen related.

To confirm that the candidate genes detected in this study are the actual causative mutations affecting CH_4_ further functional studies such as gene expression or sequencing of the region of highest interest are required. However, this was outside the scope of this paper.

### Potential Quantitative Trait Loci

Next to the search for candidate genes, we have also looked for previously detected QTLs for traits potentially related to CH_4_ production. Those QTLs were clustered in four groups of similar traits: feed efficiency, milk related, body size and health status (see Table 4). It has to be noted that in this study the estimation of CH_4_ production included an equation, in which fat-protein-corrected milk, live weight and pregnancy status are taken into account, and some of the found relationships may be present due to this fact. Alternatively, CH_4_ concentration (expressed in ppm) could be used for the association study. At this moment, however, CH_4_ production is the most widely reported trait in genetic studies regarding reduction of enteric CH_4_ emissions. For this reason we restricted our study to this trait.

Firstly, the comparison indicated an overlap between the genomic regions controlling the CH_4_ production and QTLs for feed efficiency traits (e.g. residual feed intake, feed conversion ratio, average daily gain; Table 4). The relationship between diet composition and CH_4_ production^44^ or the effect of additives reducing emission^45–49^ or dry matter intake^50–52^ is well known. It is anticipated that increased CH_4_ production leads to the loss of energy provided with feed^5,6^, and therefore more efficient cows should produce less CH_4_. Jentsch *et al*.^53^ showed that greater feed ingestion results in higher total CH_4_ production; however, CH_4_ production per kg dry matter intake decreases. Pickering *et al*.^54^ also reported the presence of a correlation between CH_4_ production and intake, while studies of^55–57^ showed that selection for cows with a low residual intake (efficient ones) results in lower CH_4_ production. Unfortunately, in this study no data was available on individual feed intake of cows and therefore we were not able to verify this statement empirically.

Secondly, regions controlling CH_4_ production were also overlapping with QTLs for traits describing various aspects of milk production (e.g. milk yield, milk protein and fat yield, milk composition; Table 4). The relationship between milk composition and CH_4_ production is particularly plausible because of common biochemical pathways between CH_4_, acetate and butyrate^58^. Furthermore, earlier studies showed that it is possible to use milk fatty acid composition to predict CH_4_ production^24,33,35–39^.

Thirdly, it was found that height, chest depth and body weight of the cow were genetically controlled by the same regions as potential QTLs for the CH_4_ production. Body characteristics such as body weight were earlier shown to be related to CH_4_ production^52,59,60^. Heavier cows are usually bigger and have a larger rumen capacity and a lower passage rate^61^, which leads to greater CH_4_ production^52^.

Finally, the QTLs detected previously for the health status of the cow (e.g. mastitis, somatic cell score, immunoglobulin G level) were also found in regions overlapping with SNPs detected in this study for CH_4_ production. Thus reports on the relationship between the health status of the animal and the direct CH_4_ production are limited. Zetouni *et al*. (2008) showed a negative genetic correlation on the health of the cows and methane production and a very low positive genetic correlation with udder health^62^. Elliott-Martin *et al*. (1997), based on breath analyses, indicated that CH_4_ could be used to diagnose ketosis. Moreover, the health status of the animal is known to affect other traits such as dry matter intake or production, and therefore is likely to affect CH_4_ production. It is likely that a sick animal produces less methane due to a lower milk production; however, methane intensity (i.e. the amount of methane produced per kg of milk) would increase. Next to QTLs related to traits indicating the health status of the cow also QTLs indicating susceptibility to illness were found in the regions important to CH_4_ production.

Based on the several traits mentioned above that share the genetic background with CH_4_ production, it may be suggested that some of the detected regions in this study have a pleiotropic effect. This knowledge is very beneficial especially in the case of production traits controlled by the same regions as CH_4_ production (i.e. assumed to be genetically correlated), which could serve as indicator traits for enteric CH_4_ production and eliminate difficult and time-consuming phenotyping. Our findings mostly match the study of Negussie *et al*.^63^, who reviewed literature on potential indirect traits for measuring CH_4_ production. Further evaluation of genetic relationships between CH_4_ and other traits is necessary to confirm relationships revealed by our study and before inclusion of CH_4_ to the breeding program can be made.

### Power of the experimental design

The Bayesian method selected to perform GWAS for CH_4_ production allows for good distinctions between SNP with large and small effects on a trait, as in each iteration a different combination of SNPs is given a large effect. Thus detected SNPs give a valuable indication for the genomic regions potentially involved in CH_4_ production in dairy cattle. This was confirmed also by bioinformatics post-analysis of detected regions with the functions of selected candidate genes and QTLs for other traits detected within those regions. However, the total genetic variance explained by significant SNPs was very low. This could be due to several possible reasons, i.e. (1) a low number of animals used in the study, (2) the accuracy of the collected phenotypes, and (3) the polygenic nature of the studied trait.

Firstly, it should be noted that the analyzed dataset was relatively small, and therefore the power of the GWAS design was too low to detect a majority of SNPs associated with CH_4_ production. Taking into account the heritability of this trait at 0.27^13^, a higher number of genotyped animals would be needed to obtain a higher percentage of genetic variance explained by the detected SNP. Therefore, the analyses of a larger dataset (for both phenotypic and genomic data) may shed light on more specific SNPs with large effects However, generating a large data set by one project is difficult due to related costs (measuring and genotyping). Therefore, generating such a dataset by combining phenotypic observations and genotypes from various experiments could be a solution producing more reliable results in the future.

Secondly, to obtain reliable GWAS results reliable phenotypes are needed. In our study we used a technique that measures CH_4_ at the AMS during milking. To verify the accuracy of the sensor used in this study we validated the used sensor against sensors used in Respiration Chambers (the standard CH_4_ measuring technique). This comparison showed a high similarity between results generated by the two sensors when used in the AMS^64^. There are no studies comparing the performance of sensors used in the present study when installed in the Respiration Chamber. Several factors could lead to inaccuracies in the collected measurements such as occasional wind in the area of AMS or cows’ head movement. These factors were not controlled in this study. To account for these arguably random effects we measured CH_4_ for the individuals in the long period of time (i.e. resulting in multiple observations per animal). The average repeatability of the analyzed phenotype was 0.25 as reported in Pszczola *et al*.^13^.

Thirdly, the greater data set and increased accuracy of the measuring method could not have been enough to explain more genetic variation if the analyzed trait was highly polygenic. In previous studies using the same methodology, but larger data sets, only 0.83% of the genetic variance was explained by SNP in GWAS on litter size in pigs^65^ and 9.5% in GWAS on teat number in pigs^66^. Based on the presented results it seems that CH_4_ production is also a highly polygenic trait and many different regions are involved in its regulation. It might not be, therefore, possible to detect all of them using GWAS.

As CH_4_ production turned out to be a very polygenic trait in application to breeding practice, it may be more advisable to use the genomic prediction approach without specifying particular SNPs as being more important than others (e.g. genomic BLUP). In fact, de Haas *et al*.^67^, Lassen *et al*.^68^ and Wilson *et al*.^69^ performed genomic prediction type analyses while searching for correlated traits. The biggest challenge for the performance of genomic prediction with sufficient, reasonable or high accuracy of the estimated genotypic values is to create an adequately large reference population, which is likely to require cooperation between several countries.

## Conclusions

This study aimed at detecting genomic regions affecting CH_4_ production in dairy cattle and showed that SNPs associated with the trait of interest may be detected. However, CH_4_ data collection poses a challenge, leading to a lower power of the experimental design and prevented detection of a high number of SNPs with a large effect on CH_4_ production. Consequently, only a small proportion of the genetic variance was explained by the SNPs. Nonetheless, the candidate QTL region on BTA 25, where three candidate genes were identified, may be considered as a genomic region regulating CH_4_ production in dairy cattle. Furthermore, the comparison of the QTL regions affecting CH_4_ production with previously reported QTLs indicated common genomic regions between CH_4_ production and traits related to feed efficiency, milk related, body size and health status. The found candidate genes were also involved in a number of metabolic processes possibly related to CH_4_ production. One of the most promising candidate genes (*PKD1*) was related to the development of the digestive tract being the environment inhabited by methanogens and the site for methane production. In general, all the evidence shows that CH_4_ production is a polygenic trait.

## Methods

All research was approved by the Local Ethical Committee for Experiments on Animals in Poznan, Poland (Decision Number: 64/2012) and performed in accordance with the “Act on the protection of animals used for scientific purpose” of the Republic of Poland, which complies with the European Union Legislation for the protection of animals used for scientific purposes.

### Phenotypes

The observations on CH_4_ production [g/d] used in this study were obtained from Pszczola *et al*.^13^, where all the detailed information on farms, measuring set-up and data processing can be found.

In short, animals available for this study were 287 Polish Holstein-Friesian cows kept on two commercial farms in Poland. This was a subset of 483 cows phenotyped for CH_4_ production and analyzed in Pszczola *et al*.^13^, of which 287 were genotyped. The CH_4_ production was measured repeatedly on Farm1 during two periods: from 2014/12/02 to 2016/02/03, and from 2016/06/01 to 2016/09/17, and on Farm2 from 2016/02/05 to 2016/03/14. Cows were milked repeatedly during the experiment, in total 25,872 CH_4_ production observations were collected for the genotyped animals.

The CH_4_ production was measured using a non-invasive Fourier Transform Infrared Spectroscopy breath analyzer (GASMET 4030; Gasmet Technologies Oy, Helsinki, Finland) during milking in AMS (Lely Astronaut A4). Concentrations of CH_4_ and CO_2_ measured during milking were converted to daily CH_4_ production in grams per day [g/d] following Madsen *et al*.^28^ and Pedersen *et al*.^70^. This calculation took into account the concentrations of CH_4_ and CO_2_, fat-protein corrected milk, live weight and duration of the pregnancy. Multiple daily outputs per cow were corrected for the diurnal variation in CH_4_ and averaged per cow per day.

### Genotypes

Cows were genotyped with the Illumina BovineSNP50 v2.0 BeadChip (Illumina Inc., San Diego, CA) at the Cattle Genetics Laboratory of the Polish Federation of Cattle Breeders and Dairy Farmers. Ear tissue samples used to extract DNA were collected in the course of a routine procedure within the breeding program. The genotyped SNPs were processed with following quality control checks: (1) being in Hard-Weinberg equilibrium, (2) having the minor allele frequency above 0.05, (3) not being monomorphic, and (4) having a call rate of above 0.95. Six cows were removed as they had the call rate below 0.9. After quality control and removing SNPs located on sex chromosomes and chromosome 0 (unassigned), 39,680 SNPs remained for the genome-wide association analysis.

### Genome-wide association

To identify regions of the genome affecting CH_4_ production, a multi-SNP genome-wide association analysis was performed with the application of the Bayesian Variable Selection method^71^. The method allows for a simultaneous estimation of the effects of all markers used in the analysis. The analysis was performed with the Bayz software^72^ on daily CH_4_ production using the model developed by Pszczola *et al*.^13^. The model was:

$$\begin{array}{c}{\bf{C}}{{\bf{H}}}_{4}=\mu +{\bf{Xb}}+{{\bf{L}}}_{{\bf{k}}}\times {\sum }_{{\bf{n}}{\boldsymbol{=}}1}^{{\bf{3}}}{\bf{D}}{\bf{I}}{{\bf{M}}}_{{\bf{i}}{\bf{j}}}+{\boldsymbol{e}}\\ {\bf{with}}\,{{\bf{L}}}_{{\bf{k}}}\times {\sum }_{{\bf{n}}=1}^{{\bf{3}}}{\bf{D}}{\bf{I}}{{\bf{M}}}_{{\bf{i}}{\bf{j}}}\,={{\bf{Z}}}_{{\bf{u}}}{{\boldsymbol{\beta }}}_{{\bf{i}}{\bf{j}}{\bf{k}}}+{{\boldsymbol{\varepsilon }}}_{{\bf{i}}{\bf{j}}{\bf{k}}},\end{array}$$

where CH_4_ stands for the daily CH_4_ production levels of a cow; µ is an n-vector equal to the mean; **Xb** is the design matrix of fixed effects of year-week of measurement and cow’s lactation number (levels 1 or 2+) fitted within the general lactation curve, which was modeled using 3^rd^ order Legendre polynomials; and ***e*** is an n-vector of random residual effects assumed to be normally distributed ${\rm{N}}(0,{\sigma }_{{\rm{e}}}^{{\rm{2}}})$. The **L**_**k**_ is a vector of individual random animal effect, which was modeled using 2^nd^ order Legendre polynomials. The mapping of marker effects is constructed as a hierarchical model on random animal effects^73^. Firstly, the model accounts for genetic variance only. Secondly, at the next level the model allows disentangling permanent environmental (Note: this accounted for repeated observations of daily CH_4_ production per cow.) and genetic variances independently for each level of 2^nd^ order Legendre polynomials. Here the **Z**_**u**_ is a matrix with dimensions *n* by *p*, with *n* being the number of genotypes and *p* being the number of SNP coded as 0, 1, 2 copies of a specific allele vector; **β**_**ijk**_ is a p-vector with the random effects of markers; and *ε*_ijk_ accounts for the permanent environmental effect assumed to be normally distributed ${\rm{N}}(0,{\sigma }_{{\varepsilon }_{{\rm{ijk}}}}^{{\rm{2}}})$.

For the marker effect the Bernoulli distribution was applied:

$$\beta  \sim \{\begin{array}{cc}N(0,{\sigma }_{{g}_{0}}^{2}) & {\rm{with}}\,\mathrm{probability}:{\pi }_{{\rm{0}}}\\ N(0,{\sigma }_{{g}_{1}}^{2}) & {\rm{with}}\,\mathrm{probability}:{\pi }_{{\rm{1}}}\end{array}$$

where for the first distribution it is assumed that the SNPs have a small effect (${\sigma }_{{g}_{0}}^{2}$); whereas in the second distribution the SNPs are assumed to have a large effect, which explains a large part of variance (${\sigma }_{{g}_{1}}^{2}$) of analyzed traits. In this study, a prior of *π*_1_ = 0.001 was selected, thus on average only 1 in 1,000 SNPs was in the second distribution in each cycle. This resulted in only ~38 SNPs per cycle to have a large effect on the traits. The posterior means were calculated with 500k MCMC iterations with burn-in of 5k iterations to secure that all the SNPs were used^65,66,74^. Selecting a stringent prior provides a more precise distinction between SNPs with large and small effects on the trait^66,75^. If the SNP was not genotyped for a certain animal then Bayz assigned an average genotype to that position.

### Identification of significant SNPs

The Bayes Factor (BF) was calculated for each SNP to determine the significant associations:

$${\rm{BF}}=\frac{{\hat{p}}_{i}/(1-{\hat{p}}_{i})}{{\pi }_{1}/{\pi }_{0}},$$

where *π*_1_ and *π*_0_ are the prior probabilities and ${\hat{p}}_{i}$ is the posterior probability of the fraction of times the SNP was in the distribution with a large effect. Following the definitions of Kass and Raftery^20^, the SNPs with BF > 30 are described as a “very strong” association and with BF > 150 as “decisive”. The variance explained by significant SNPs was estimated as a fraction of the total genetic variance explained by all SNPs.

To confirm the potential QTL regions, also the linkage disequilibrium (LD) measured by r^2^ was estimated in Haploview^21^ between the SNPs detected on one BTA and not further from each other than 500 kbp. The candidate gene search was performed with the BIOMART software available in Ensembl Bos Taurus UMD 3.1^32^ by entering the position of a possible QTL region or one of the most significant SNPs with ±500 kbp. To limit the number of QTLs to the most promising as candidate genes for daily CH_4_ production the BIOMART database was also used to study Gene Ontology Terms (GO Terms) of those QTLs. Furthermore, the Cow QTL database of the Animal Genome project^20^ was used to find previously detected QTLs within the most promising regions detected here for daily CH_4_ production. This was done analogically as for the candidate gene search, i.e. by entering the position of a possible QTL region or one of the most significant SNPs with ±500 kbp.
